# Understanding Dengue Virus Capsid Protein Interaction with Key Biological Targets

**DOI:** 10.1038/srep10592

**Published:** 2015-07-10

**Authors:** André F. Faustino, Ivo C. Martins, Filomena A. Carvalho, Miguel A. R. B. Castanho, Sebastian Maurer-Stroh, Nuno C. Santos

**Affiliations:** 1 Instituto de Medicina Molecular, Faculdade de Medicina, Universidade de Lisboa, Lisbon, Portugal; 2 Bioinformatics Institute (BII), Agency for Science, Technology and Research (A*STAR), Singapore; 3 School of Biological Sciences (SBS), Nanyang Technological University (NTU), Singapore

## Abstract

Dengue virus (DENV) causes over 500,000 hospitalizations and 20,000 deaths worldwide every year. Dengue epidemics now reach temperate regions due to globalization of trade and travel and climate changes. Currently, there are no successful therapeutic or preventive approaches. We previously developed a peptide drug lead, pep14-23, that inhibits the biologically relevant interaction of DENV capsid (C) protein with lipid droplets (LDs). Surprisingly, pep14-23 also inhibits DENV C interaction with very low-density lipoproteins (VLDL). We thus investigated the similarity between the proposed DENV C molecular targets in LDs and VLDL, respectively, the proteins perilipin 3 (PLIN3) and apolipoprotein E (APOE). APOE N-terminal and PLIN3 C-terminal regions are remarkably similar, namely APOE α-helix 4 (APOEα4) and PLIN3 α-helix 5 (PLIN3α5) sequences, which are also highly superimposable structurally. Interestingly, APOE α-helical N-terminal sequence and structure superimposes with DENV C α-helices α1 and α2. Moreover, the DENV C hydrophobic cleft can accommodate the structurally analogous APOEα4 and PLIN3α5 helical regions. Mirroring DENV C-LDs interaction (previously shown experimentally to require PLIN3), we experimentally demonstrated that DENV C-VLDL interaction requires APOE. Thus, the results fit well with previous data and suggest future drug development strategies targeting the above mentioned α-helical structures.

Dengue virus (DENV) causes a mosquito-borne disease that leads to over half a million hospitalizations and 20,000 deaths worldwide every year^1^,^2^,^3^,^4^. A recent estimative puts the global toll in 390 million people infected per year^5^, roughly three times higher than earlier projections^1^,^3^,^4^,^6^. The infection by DENV can have several clinical manifestations, ranging from essentially asymptomatic to the well-known dengue hemorrhagic fever and the often fatal dengue shock syndrome^1^,^3^,^4^,^5^,^6^,^7^,^8^. Importantly, epidemiological data suggest that people who have been previously exposed to one of the four dengue virus serotypes are, in a subsequent infection by another serotype, at a greater risk of developing more severe forms of the disease, including the often fatal hemorrhagic fever^9^. DENV serotypes are now spreading further due to the globalization of trade and travel, increasing the range and frequency of dengue epidemics^1^,^5^,^10^,^11^,^12^,^13^,^14^,^15^,^16^,^17^. Furthermore, globalization and climate changes have also fuelled the geographical expansion of the dengue mosquito vectors, *Aedes albopictus* and *Aedes aegypti*^2^,^5^,^10^,^11^,^12^,^18^,^19^. Their area of distribution now includes not only tropical and subtropical regions of the planet, but also temperate areas in USA^12^ and Europe^11^,^19^, where three local outbreaks of the disease have occurred since 2010^13^,^14^,^15^,^16^,^17^. Importantly, even in temperate climates, *Aedes* spp. mosquitoes eradication attempts have been largely unsuccessful^2^,^19^. Dengue is the world’s fastest-growing tropical disease, for which there are no effective and specific treatments or commercial vaccines^1^,^20^, partially due to the lack of knowledge on basic aspects of the viral life cycle^21^. The development of an effective drug or therapy against dengue is thus a major priority.

For this purpose key insights can be gained from comparing DENV with other closely related viruses. DENV belongs to the *Flavivirus* genus and *Flaviviridae* family^21^, a taxon that includes other major human pathogens, such as the yellow fever virus, West Nile virus and hepatitis C virus, among others^21^,^22^. These viruses share common life cycle features and similar virion structure^21^, with homologous proteins presenting highly conserved regions^21^,^23^,^24^. The viral particle is relatively simple: a lipid bilayer, where the envelope and membrane proteins are located, surrounds a nucleocapsid where a positive sense single-stranded genomic RNA forms a complex with multiple copies of the capsid (C) protein^25^. In addition to these three structural proteins, DENV presents seven non-structural proteins^21^. Despite their name, structural proteins possess a number of other roles in the virus life cycle. DENV C is an 100 amino acid residues homodimeric protein, containing four α-helical regions and an intrinsically disordered N-terminal domain^23^,^26^,^27^ (see Supplementary Fig. A online). This protein is a potential drug target against DENV infection^28^ due to its interaction with host intracellular lipid droplets (LDs), essential for viral replication^29^, and with very low-density lipoproteins (VLDL)^23^,^30^,^31^, through which it may prompt the formation of lipoviroparticles^31^ (described below).

We have previously studied DENV C-LDs interaction using different biophysical techniques and viral replication assays^23^,^30^. The data allowed us to show that DENV C binding to LDs is strong, specific, dependent on the high intracellular potassium concentrations and using perilipin 3 (PLIN3, also known as TIP47^32^) as the major ligand on LDs surface^30^. Secondly, we identified the DENV C amino acid residues involved in this interaction^23^. This revealed that both the DENV C central hydrophobic α2-α2′ patch and a conserved N-terminal segment are involved in the binding to LDs^23^. These results allowed us to design pep14-23, a peptide based on part of the DENV C N-terminal domain and that acquires α-helical structure in the presence of negatively charged phospholipids^33^ being able to inhibit DENV C-LDs binding (an interaction required for viral replication), paving the way for new drug development approaches against dengue^23^,^28^,^33^.

We also studied DENV C interaction with human lipoproteins^31^, since the closely related hepatitis C virus can form highly infectious lipoviroparticles through the conjugation of the virion with host plasma lipoproteins^34^,^35^. We found that DENV C interacts strongly and specifically with VLDL, but not with low density lipoproteins (LDL)^31^. A major difference between these two plasma lipoproteins is that apolipoprotein E (APOE) is typically found in the VLDL surface, while it is almost totally absent in LDL^36^,^37^. Strikingly, APOE is an α-helical protein^38^ that is structurally similar to the LDs surface protein PLIN3^39^. This parallel, together with the absence in LDL, hints that APOE may be the DENV C molecular target on VLDL surface^31^. Supporting this hypothesis, the DENV C-VLDL interaction also requires potassium ions as in the case of DENV C-LDs binding and, most importantly, it is also inhibited by pep14-23, suggesting a common mode of action^31^.

Here, taking all the above into account, we aimed at evaluating if DENV C interaction with LDs and VLDL may involve specific and similar sequences/structures of, respectively, PLIN3 and APOE. Our data support the hypothesis, suggesting also that the DENV C hydrophobic cleft can accommodate the structurally similar APOE α-helix 4 and PLIN3 α-helix 5 regions in a direct interaction with DENV C α-helices 1 and 2, fitting well with previous data^23^,^30^,^31^ and suggesting future avenues of research by targeting the helical structures proposed to be involved in these interactions.

## Results

**Figure 1:**
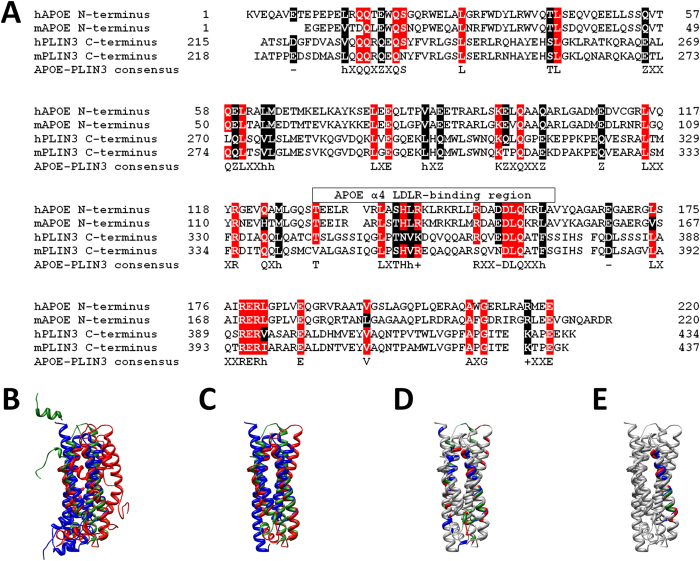
Sequence and structure similarity between apolipoprotein E and perilipin 3. (**A**) Conserved amino acid residues between APOE N-terminal and PLIN3 C-terminal regions. Red stands for strictly conserved residues and black for stereochemically and/or functionally identical residues. In the APOE-PLIN3 consensus line, “h”, “+”, “–” and “Z” stand respectively for hydrophobic, cationic, anionic and E or Q (glutamate or glutamine)^63^. The consensus includes the APOE α4 region (between residues T130 and A160), corresponding to the APOE LDLR-binding region^38^,^40^,^41^,^42^,^43^. (**B**) APOE α-helix 4 (APOEα4) and PLIN3 α-helix 5 (PLIN3α5) present highly similar structures, as shown by superimposing the tridimensional lipid-free structures of the full length hAPOE (red) and the mAPOE N-terminus (green) with the mPLIN3 C-terminus (blue). (**C**) APOE superimposition with PLIN3 structures occurs mostly via their common and extremely similar four-helix-bundle motif, as previously reported^39^. (**D**) Amino acid residues conserved between APOE and PLIN3 are highlighted according to the same color scheme as in (**B**) but with the non-conserved residues colored gray. APOEα4 and PLIN3α5 align particularly well in terms of sequence and structure. (**E**) Superimposition of hAPOE, mAPOE and mPLIN3 structures highlighting the residues conserved both in terms of sequence and structure (the color scheme is as described in B but showing in gray all other residues). These residues are exclusively located in the APOEα4 and in the PLIN3α5 superimposing helices. The Cα RMSD of hAPOEα4 and mAPOEα4 compared against mPLIN3α5 are 2.05 Å and 2.72 Å, respectively, within the same degree of variation of hAPOE and mAPOE α4 helices (average Cα RMSD of 2.14 Å). Taking into account the previously documented DENV C binding to VLDL^31^ and LDs^23^,^29^,^30^, the strong resemblance between APOEα4 and PLIN3α5 suggests that these highly similar α-helical regions may act as DENV C molecular targets on VLDL and LDs, respectively.

We originally demonstrated that DENV C is able to specifically bind VLDL, but not LDL^31^. This may be due to the differences in protein composition between VLDL and LDL. The former present APOE among their protein components, being essentially absent in LDL^36^,^37^. APOE may therefore be the molecular target of DENV C on the VLDL surface^31^. This is also supported by APOE structural similarity with PLIN3^38^,^39^, which is found in the LDs surface, acting as ligand for DENV C^30^. As such, to investigate a possible role for APOE in the VLDL interaction with DENV C, the amino acid sequence of PLIN3 proteins of both human (hPLIN3) and mouse (mPLIN3) were compared with the APOE sequences of human (hAPOE) and mouse (mAPOE). The comparison of the N-terminal region of APOE (first 220 residues) with the C-terminal region of PLIN3 (last 220 residues) reveals several matching motifs of conserved regions Fig. 1A). These common sections of PLIN3 and APOE may thus be involved in similar protein-protein specific interactions, namely in the interaction with DENV C, especially if the structure of those sections is also conserved.

Therefore, to evaluate the structural relevance of the identical residues on PLIN3 and APOE, the three-dimensional lipid-free structures of hAPOE, mAPOE and mPLIN3 were superimposed (Fig. 1B–E). hAPOE and mAPOE structures co-localize well in space, especially regarding a stretch of 143 conserved residues that share a similar fold and display an average Cα root-mean-square deviation (RMSD) of 2.73 Å (Fig. 1D). The comparison of both APOE structures with mPLIN3 also shows a strong similarity (Fig. 1B), especially in their four-helix-bundle motif (Fig. 1C), as noticed by other authors^39^.

In addition to the resemblance between hAPOE and mAPOE, as well as their overall structural similarity to mPLIN3, some key insights can be gained from more closely looking at mPLIN3 and hAPOE structural superposition. In particular, hAPOEα4 (see Supplementary Fig. S1B online) includes the functionally relevant LDL receptor (LDLR)-binding region^38^,^40^,^41^,^42^,^43^ (Fig. 1A) and superimposes particularly well with mPLIN3α5 (see Supplementary Fig. S1C online). In fact, mPLIN3α5 and hAPOEα4 are the only sections of these proteins that are conserved in terms of amino acid sequence (Fig. 1A) and that also co-localize well in space (Fig. 1E), with a considerably low average Cα RMSD of 2.05 Å for a 23 residues long superimposing region. This compares very well with the 2.72 Å Cα RMSD of the corresponding 23 amino acids homologous regions of mouse and human APOE protein structures.

It could be argued that the APOEα4/PLIN3α5 structural similarity could be due to both being within a roughly equally-sized α-helix. However, in addition to the structure similarity, similar amino acid sequences were also found. To evaluate the significance of this motif similarity, the frequency and identity of the proteins where this motif could be naturally found was determined. Additionally, the likelihood of the motif to occur by chance was tested by using a non-uniform Bernoulli model in a large random database^44^ while searching their consensus sequence motifs via the ScanProsite web server from ExPASy^45^ (Table 1). Taking the generic AP1 motif identified in Table 1 ([ALVIM]-X-[STCNQDEH]-[STCNQDEH]-[ALVIM]-[RK]-X(10)-[STCNQDEH]-D-L-Q-X(2)-[ALVIMFYW]) and searching for it in the UniProtKB/Swiss-Prot database, 40 matching protein sequences were identified, while only 11 would be likely to appear by random chance. From these 40 sequences, 25 are either PLIN3 or APOE sequences. Slightly restricting this motif to the AP2 motif of Table 1 ([ALVIMFYW]-X-[ST]-[NH]-[ALVIMFYW]-[RK]-X(10)-[NQDE]-D-L-Q-X(2)-[ALVIMFYW]), all protein hits that are neither APOE nor PLIN3 are excluded. Most importantly, all the 25 APOE and PLIN3 hits are kept, while only 1 hit would be expected to occur by chance. Further restricting the motif from AP3 to AP8 sequence motif (our original consensus motif generated from the alignment described in Fig. 1A), not only all random occurrences are removed, but most APOE and PLIN3 hits are also preserved (22 out of the 25 initially found). This APOEα4 alignment with PLIN3α5 is thus remarkable within the overall sequence and structure, since it even includes similar orientations of the amino acid side chains. Given the high APOEα4/PLIN3α5 similarity and the previously documented DENV C binding to VLDL^31^ and LDs^23^,^29^,^30^, APOEα4 and PLIN3α5 may be the DENV C specific targets in VLDL and LDs, respectively.

Assuming APOEα4 and PLIN3α5 as the DENV C-binding domains, this implies that further studying their sequences and structures (especially regarding the similarities they may possess) may reveal important aspects of their mode of action, including their possible interaction mechanism with DENV C. Given the interest in APOEα4 function and the wealth of information on APOE complete protein structure (see Supplementary Fig. S2A online), the currently known role of APOEα4 was analyzed within the context of the overall APOE structure (see Supplementary Fig. S2 online)^38^. Some major points should be taken into consideration. First of all, APOEα4 is physiologically relevant for protein-protein interactions since it is responsible for APOE binding to LDLR^40^,^41^,^43^. Secondly, not only APOEα4 and PLIN3α5 are strikingly similar (Fig. 1), but also APOE and PLIN3 localize at the surface of their respective lipid systems (VLDL and LDs)^38^,^46^. Third, these two lipid systems are extremely alike in terms of composition and structure^36^,^37^,^46^,^47^. As such, the conserved regions of these proteins may adopt similar conformations when located in their natural lipid systems. Studying APOE structure may thus give important clues on the understanding of APOEα4 and PLIN3α5 biological functions.

APOE experimental structural data (see Supplementary Fig. S2 online) reveals that APOEα4 interacts directly with APOE α-helical N-terminal region (APOEαN, residues K1-S44, identified in Supplementary Fig. S2B online)^38^. Importantly, APOE structure should not be considered static, as within the lipoprotein physicochemical environment the four-helix-bundle may adopt an open structure (see Supplementary Fig. S2C online)^38^. In either case, APOEα4 remains quite close to APOEαN (see Supplementary Fig. S2D online) when it is within lipoproteins (see Supplementary Fig. S2C online)^38^. Thus, the APOEαN section, containing APOE N-terminal α-helices αN2 (hAPOEαN2) and α1 (hAPOEα1), is relevant for hAPOEα4 function and deserves a closer inspection (see Supplementary Fig. S1B online for details regarding the nomenclature of hAPOE secondary structure elements).

**Figure 2:**
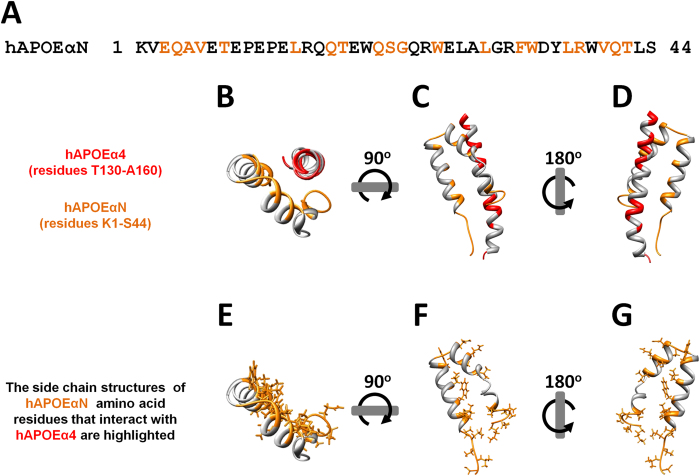
hAPOE α-helical N-terminal region interacts with hAPOEα4 through conserved residues. (**A**) Sequence of hAPOE α-helical N-terminal region (hAPOEαN, residues K1 to S44), which interacts with hAPOEα4^38^. Highlighted in orange are the hAPOEαN residues that are within 5 Å of hAPOEα4 (which is similar to PLIN3α5, as shown in Fig. 1F). (**B**–**D**) Different ribbon views of hAPOEαN and hAPOEα4. In these views, hAPOEαN residues within 5 Å of hAPOEα4 are colored in orange and hAPOEα4 residues conserved between hAPOEα4 and mPLIN3α5 are colored in red. (**E**–**G**) hAPOEαN ribbon views, showing the side chains of the residues within 5 Å of hAPOEα4 colored in orange. Proteins that interact with the hAPOEα4 region may do so via a conformational arrangement similar to hAPOEαN structure.

hAPOEαN sequence and structure are shown in Fig. 2, highlighting in orange the amino acid residues that are within 5 Å of hAPOEα4 (the hAPOE domain structurally analogous to mPLIN3α5; Fig. 1F). In the same figure, hAPOEα4 residues conserved in terms of sequence between APOE and PLIN3 are highlighted in red (Fig. 2B–D). By observing them more closely, it becomes clear that hAPOEαN and hAPOEα4 interact with each other even when hAPOE is within the lipoprotein, as previously reported^38^. Other proteins, such as the LDLR protein family, bind to hAPOEα4^38^,^40^,^41^,^42^,^43^. APOEα4 has therefore the crucial ability to mediate protein-protein interactions. Looking at the APOEαN and APOEα4 closely packed structure (Fig. 2B–D and Supplementary Fig. S2, online), it is expectable that proteins which interact with the APOEα4 region may adopt a conformation similar to APOEαN (Fig. 2E–G).

**Figure 3:**
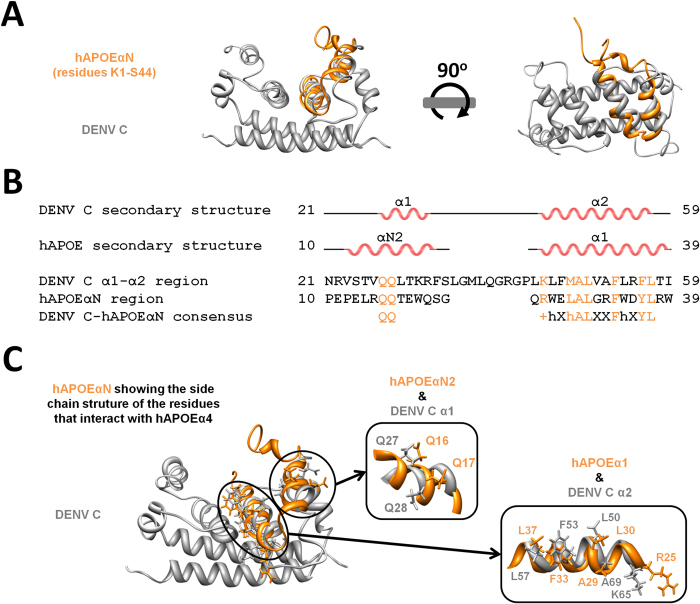
hAPOEαN structure superimposes with DENV C α-helices α1 and α2 backbone and side chains. (**A**) DENV C structure (gray) superimposed with hAPOEαN α-helices (orange), showing that hAPOEαN2 and hAPOEα1 α-helices superimpose well with the backbone structure of DENV C α1 and α2 helices. (**B**) Looking at these α-helices sequence alignment and taking into account the structural superposition, it can be noticed that several functionally relevant amino acid residues are in similar positions of the sequences, namely the region “ +hXhALXXFhXYL” (DENV C α2 and hAPOEα1). (**C**) A closer inspection of the side chains orientation of the functionally relevant amino acid residues of both proteins shows that their side chain structures also superimpose. Additionally, the side chains of functionally relevant residues of hAPOEαN^38^ and DENV C^23^ display a similar spatial orientation.

**Figure 4:**
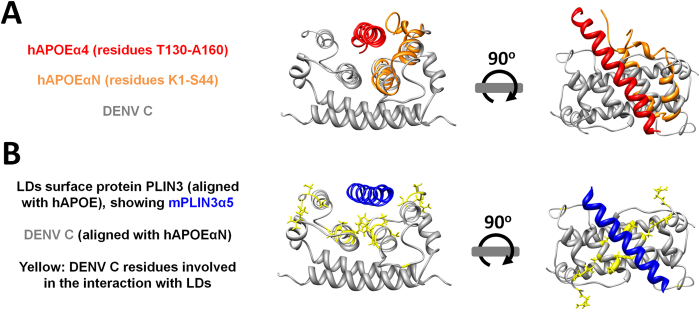
DENV C hydrophobic cleft can accommodate the structurally analogous hAPOEα4 and mPLIN3α5 α-helical regions. (**A**) DENV C (gray) hydrophobic cleft (formed by the α1-α2-α2′-α1′ regions) is involved in the interaction with LDs^23^ and is suggested to also play a role in DENV C interaction with VLDL^31^. As shown in Fig. 3, DENV C α1 and α2 helices fit structurally with the hAPOEαN helices (orange). If hAPOEα4 (red) is displayed as when interacting with hAPOEαN^38^, it is clear that the entire APOEα4 α-helical region fits in the DENV C hydrophobic pocket. (**B**) Structurally aligning onto the previous figure the PLIN3α5 (blue) structure (which superimposes with APOEα4, as depicted in Fig. 1) shows that PLIN3α5 also fits the same pocket of DENV C structure. The DENV C residues involved in PLIN3-mediated DENV C interaction with LDs^23^,^30^ are colored in yellow, showing that they would clearly be affected if PLIN3 binds to DENV C via the hydrophobic pocket, as proposed. Overall, this fits well with previous data showing DENV C interaction with LDs^23^,^29^,^30^ and VLDL^31^.

Considering that (i) DENV C interacts with APOE-containing VLDL^31^ and with LDs via PLIN3^30^, (ii) PLIN3 and APOE are extremely similar^39^, (iii) mPLIN3α5 is extremely similar to hAPOEα4, (iv) hAPOEα4 is functionally relevant for protein-protein interactions, and (v) hAPOEαN interacts directly with hAPOEα4, we hypothesize that the proposed hAPOE-DENV C interaction^31^ may occur via hAPOEα4 binding to the DENV C domain structured similarly to hAPOEαN. Thus, DENV C and hAPOEαN structures were compared in detail (Fig. 3). As shown in Fig. 3A, the hAPOE N-terminal α-helices N2 (hAPOEαN2) and 1 (hAPOEα1) superimpose perfectly with DENV C α-helices 1 and 2, respectively, with an overall average Cα RMSD of 1.68 Å for a 20 amino acid residues long superposition (please consult Supplementary Fig. S1 online for hAPOE motifs nomenclature). Therefore, DENV C α-helices 1 and 2 mimic the conformation of hAPOEαN. Moreover, looking at the sequence alignment of these APOE and DENV C domains (Fig. 3B) within the context of the structural superposition (Fig. 3A), we observe that several functionally relevant residues are in similar positions of the sequences (Fig. 3B,C). This alignment is prominent at DENV C α2 (from residues K45 to L57) and APOEα1 (from R25 to L37), yielding a 13 amino acid residues consensus: “+hXhALXXFhXYL” (“h” and “+” stand, respectively, for hydrophobic and cationic residues). The same stretch of similar residues is crucial for DENV C interaction with LDs via the surface protein PLIN3^23^,^29^,^30^. Remarkably, this sequence and backbone structure superposition of APOEαN2 and APOEα1 with DENV C helices α1 and α2, respectively, is also extended to the side chains of specific amino acid residues of both proteins (Fig. 3C). Most importantly, the DENV C α2 residues that superimpose both in the backbone and side-chains are involved in the interaction with lipid droplets^23^ via PLIN3^30^. Thus, overall, the hAPOEαN residues that bind hAPOEα4 are very similar to functionally relevant DENV C residues, displaying comparable backbone and side-chain structural orientation and sequence (Fig. 4).

To determine the likelihood of a random simultaneous occurrence of the 13 amino acid residues motif [RK]-[LMW]-X-[LM]-A-[FL]-X(2)-F-[LFW]-[DRK]-[FVY]-L (named AF8 in Table 2) present in APOE and DENV C, the ExPASy ScanProsite web server^45^ was employed to evaluate the frequency of such a motif in the UniProtKB/Swiss-Prot protein database (Table 2). Interestingly, this motif is highly conserved among dengue virus capsid proteins and primate APOE proteins, supporting a common role of these regions of APOE and DENV C. It is also not a random occurrence, since 26 APOE and DENV C conserved sequences appear where none is expected to occur just by chance. Importantly, this is a region identified as essential for DENV C interactions with LDs^23^,^29^, being conserved among the four dengue virus serotypes^48^,^49^. Given the previously reported sequence homology between the capsid proteins of other mosquito-borne *Flavivirus*^23^, the more generic motifs common to APOE and DENV C proteins, such as the AF6 motif, were also studied by searching them in the database (Table 2). As expected, when searching for a more generic and less restrictive motif, other protein sequences were found besides APOE and DENV C proteins. Interestingly, several of these were C protein sequences of other *Flavivirus*. Remarkably, 33 sequences were found to contain the AF6 motif (comprising 4 APOE protein hits, and 22 capsid proteins from DENV strains and 7 other *Flavivirus*). According to the statistical algorithm, the likelihood of this being a random protein hit occurrence in the database is null. Making the motif more generic (sequences AF5 to AF1 of Table 2) does not increase substantially the number of random hits of proteins that are neither APOE nor *Flavivirus* capsid proteins. The most generic motifs tested, AF1 and AF2, generate 61 and 42 hits, respectively. Although these are very generic motifs, the majority of the hits (33 hits) are still from APOE or flaviviruses C proteins. Overall, the appearance of other flaviviruses C proteins in the UniProtKB/Swiss-Prot search (Table 2) supports a generic role for these APOE-like motifs across related viruses. Most importantly, together with the statistical analysis performed, this also demonstrates that the structural and sequence similarities between APOE and DENV C described previously are likely to be functionally relevant and deserve to be investigated in detail. Most importantly, the data gathered clearly suggest APOE as being the molecular target of DENV C on the VLDL surface.

**Figure 5:**
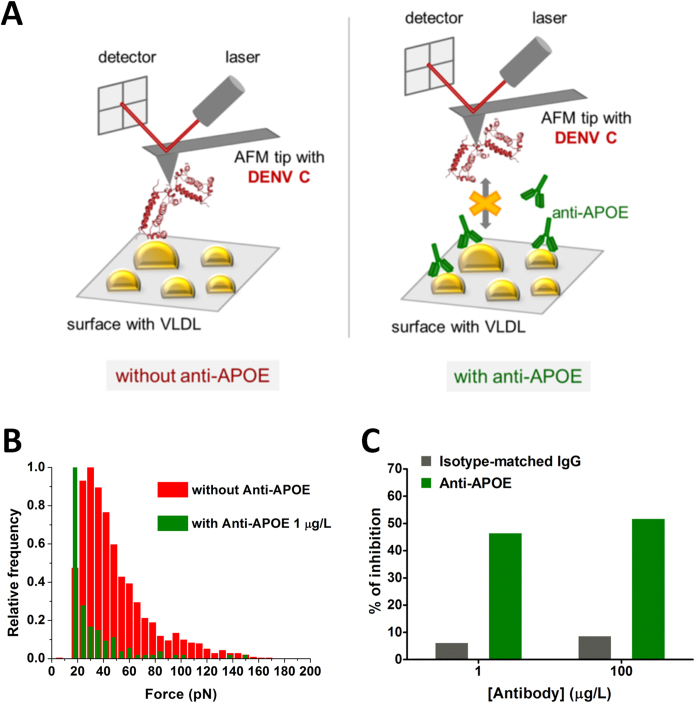
DENV C protein binds specifically to APOE on VLDL. (**A**) Schematic representation of the AFM-based force spectroscopy inhibition assay. DENV C, covalently attached to the AFM tip, binds to VLDL adhered to the mica surface, in buffer, in the absence of the anti-APOE antibody (left panel); however, DENV C-VLDL interactions are inhibited by the presence of anti-APOE (right panel). Binding events are measured through the change on the deflection of the AFM cantilever, which is detected by a laser pointed on the backside of the cantilever and reflected on a photodetector. (**B**) Force-rupture histograms for the DENV C-functionalized AFM tips interacting with VLDL in the absence (red) or presence (green) of 1 μg/L anti-APOE antibody. (**C**) According to the percentage of (un)binding events, the percentage of inhibition of the DENV C-VLDL binding in the presence of anti-APOE antibody is 46.9% at 1 μg/L and 52.2% at 100 μg/L (green bars). Controls were performed with the same concentrations of an isotype-matched antibody, yielding a percentage of inhibition of just 6.6% and 9.1%, respectively (gray bars). Looking solely on the stronger interaction forces typical of specific binding events (>25 pN), the effect of anti-APOE at 1 μg/L becomes even clearer, with 75.2% DENV C-VLDL binding inhibition. All experiments (and respective controls) were performed in triplicate with the same acquisition settings, leading to approximately 5000 single rupture-force measurements for each histogram.

As such, following previously established procedures^23^,^30^,^31^,^50^, atomic force microscopy (AFM)-based force spectroscopy studies were employed to determine the role of APOE on DENV C-VLDL binding. Briefly, tapping on VLDL with AFM tips where DENV C was covalently attached allowed measuring the frequency (or probability) of DENV C-VLDL (un)binding events, as well as the respective binding force, in the presence and in the absence of a specific anti-APOE antibody, as schematized in Fig. 5A. The resulting rupture force histograms depict a substantial reduction of the strong and specific interactions between DENV C and VLDL in the presence of the anti-APOE antibody at 100 μg/L or 1 μg/L (Fig. 5B). Such a reduction of the frequency of DENV C-VLDL (un)binding events is not observed in the presence of either 100 μg/L or 1 μg/L of an isotype-matched antibody not specific for APOE. Comparing DENV C binding to VLDL in the absence of antibodies and in the presence of the lowest concentration tested (1 μg/L of the anti-APOE specific antibody) shows that the presence of the antibody causes a 46.9% inhibition of DENV C-VLDL binding (defined as the reduction of the frequency or probability of binding events; Fig. 5C). At 100 μg/L of anti-APOE antibody, a 52.2% inhibition was observed. Control experiments with VLDL incubated with 1 μg/L or 100 μg/L of the isotype-matched antibody yielded only a 6.6% or 9.1% reduction of the frequency of (un)binding events, respectively (Fig. 5C). The effect of anti-APOE on the reduction of the specific (un)binding events becomes even clearer if one takes into consideration a force threshold at 25 pN, considering as specific binding events those above this value. In this region of stronger and specific forces^30^,^31^, the percentage of inhibition by 1 μg/L anti-APOE is 75.2% (Fig. 5B). The weak interactions remaining after anti-APOE treatment are essentially related to unspecific binding events. In the absence of the antibodies, the force necessary to break the DENV C-VLDL binding is 29.7 ± 0.4 pN and 52.1 ± 4.4 pN (values are mean ± s.e.m.), respectively for the 1^st^ and 2^nd^ Gaussian peaks of the histogram. In the presence of anti-APOE, a weaker peak emerges at approximately 20 pN. This weaker force is typical of unspecific weak binding events, as observed in previous studies of DENV C binding to LDs^30^ and VLDL^31^. These results further demonstrate that APOE is the molecular target of DENV C on the VLDL surface, as discussed ahead.

## Discussion

Taking all of the above into account, given that APOEαN binds specifically to APOEα4^38^, it is thus anticipated that DENV C, namely through its α1 and α2 helices (structurally analogous to APOEαN), is able to perform a similar function, binding to APOEα4. This would explain its documented ability to bind VLDL (rich in APOE) but not to LDL^31^. With this in mind, the overall meaning of the sequence and structure similarities between APOE, PLIN3 and DENV C proteins were further investigated. As shown in Fig. 4, APOE and DENV C structures were compared taking into account APOEαN ability to interact with APOEα4 (Fig. 2A and Supplementary Fig. 2 online) and APOEαN similarity to DENV C α1 and α2 helices (Fig. 3). Briefly, first the structures of APOEαN with DENV C were aligned as described in Fig. 3. Next, these aligned structures were superimposed with the complete APOE structure. For clarity, only the APOEα4 helix is then displayed (as described in Fig. 2). A perfect fit of APOEα4 into the DENV C hydrophobic cleft is immediately observed, as shown in Fig. 4A. Finally, as described for Fig. 1, PLIN3 is then superimposed onto APOE. The final image is shown in Fig. 4B, showing that PLIN3α5 also fits within the DENV C α1-α2-α2′-α1′ pocket. Therefore, from Fig. 4 it is clear that DENV C hydrophobic α1-α2-α2′-α1′ pocket (*i.e.*, formed by its α2-α2′ interface and the α1 and α1′ structural domains) can accommodate APOEα4 and PLIN3α5 α-helical regions. To stress the relevance of these matches, in Fig. 4B we also highlighted in yellow the side chains of the residues of DENV C reported to be affected by the interaction with LDs^23^. In such an arrangement, the LDs surface protein PLIN3 would be able to interact with DENV C relevant residues via its PLIN3α5 α-helix, fitting well with previous experimental information^23^,^30^.

The data presented here indicate that PLIN3α5, as well as APOEα4, can interact with the α2 and α1 domains of DENV C (Fig. 4). This agrees well with the documented DENV C direct interaction with LDs^23^,^30^, which involves residues of those α-helices (as well as N-terminal residues^23^) and the LDs surface protein PLIN3^30^. Interestingly, the functionally relevant APOEα4 region^38^,^40^,^41^,^42^,^43^ is similar in structure and sequence to PLIN3α5 (Fig. 1), while APOEαN and DENV C α1-α2 are perfectly superimposing structures (Fig. 3). APOEα4 and PLIN3α5 fill the three-dimensional hydrophobic pocket of DENV C in such an elegant manner that conserved DENV C residues fundamental to DENV C-LDs interaction^23^ would be able to promote that binding (Fig. 4).

The results presented here point to APOE as the main protein ligand of DENV C on the VLDL surface (Fig. 5). The inhibitory effect of an anti-APOE antibody on DENV C-VLDL interaction is demonstrated by single-molecule AFM-based force spectroscopy data. A decrease on the binding force and on its probability to occur is observed in the presence of the antibody. The effect of anti-APOE on the reduction of the specific (un)binding events becomes even clearer if one takes into consideration a force threshold at 25 pN, considering as unspecific binding events those below this value. In this region of stronger and specific forces^30^,^31^, the percentage of inhibition is 75.2% (Fig. 5B). Almost only unspecific weak binding events remain on anti-APOE treated VLDL. Importantly, DENV C-VLDL interaction is just slightly affected by the presence of an isotype-matched antibody (Fig. 5C). All this clearly supports APOE as the VLDL surface ligand of DENV C. It could be reasoned that, as APOE can also be found in LDL (although to a quite lesser extent than on VLDL)^51^,^52^,^53^, it could also allow for DENV C-LDL binding. However, this does not occur, as determined experimentally^31^. The percentage of LDL particles that contain APOE is below 4.4%^54^,^55^,^56^,^57^. As such, when using single-molecule AFM-based force spectroscopy to study how a single DENV C molecule interacts with an LDL particle, there is a <5% chance of encountering an LDL particle containing APOE. Even for those particles that do have APOE, stochastically, a binding event would be established only with a subfraction of them. As a result, no statistically significant binding would be expectable. This is indeed what we previously observed experimentally for DENV C binding to LDL^31^.

To conclude, the similarities between DENV C interaction with LDs and VLDL are striking. All these interactions require potassium ions^30^,^31^ and, as demonstrated here, the PLIN3-mediated LDs and the APOE-mediated VLDL interaction with DENV C are also considerably similar in terms of functionally relevant structures and amino acid sequences. Overall, we propose that DENV C-VLDL binding involves APOEα4 residues in a similar way as DENV C-LDs interaction requires PLIN3α5 residues. This explains how DENV C binding to VLDL can be inhibited by pep14-23^31^, a peptide originally designed to prevent the PLIN3-mediated DENV C interaction with LDs^23^,^28^. Noteworthy, an APOE-derived peptide corresponding to the APOEα4 region highlighted here was also found to inhibit viral entry of the closely related hepatitis C virus^58^. Importantly, the general concept of viral proteins mimicking host protein structures for interactions has also been observed before for other viruses, for example with influenza NS1 acting as histone mimic for hPAF1C (human PAF1 transcription elongation complex) interaction^59^. These findings also suggest future avenues of research aiming at simultaneously targeting DENV C interactions with VLDL and LDs by focusing on the inhibition of these specific protein-protein interactions via rational drug design. The inhibition of these interactions would also be a good tool to study the potential biological relevance of APOE for the dengue virus life cycle. Finally, given the occurrence of APOE-like motifs in several other flaviviruses capsid proteins, as reported here, this approach may thus be of potential use to target closely related viruses such as Japanese encephalitis virus, St. Louis encephalitis virus, Murray valley encephalitis virus, Kunjin virus and West Nile virus.

## Methods

### Sequence alignment and consensus regions between apolipoprotein E and perilipin 3

Protein sequences were obtained from the National Center for Biotechnology Information Protein database (www.ncbi.nlm.nih.gov/protein/) with the reference numbers stated ahead in parenthesis. Human (*Homo sapiens*) and mouse (*Mus musculus*) proteins are identified by the prefixes “h” and “m”, respectively. In apolipoproteins E (hAPOE, NP_000032.1; mAPOE, NP_033826.2), we discarded the initial 18 amino acids of each sequence, which correspond to the transmembrane signal peptide that is cleaved after translation within the endoplasmic reticulum (according to the NCBI information retrieved from the SignalP 4.0 algorithm^60^). We compared the N-terminal APOE proteins (residues 1 to 220) that align with C-terminal regions of human (hPLIN3, NP_005808.3, residues 215 to 434) and mouse (mPLIN3, NP_080112.1, residues 218 to 437) perilipin 3 (PLIN3) proteins by performing a multiple sequence alignment using Clustal W2 (www.ebi.ac.uk/Tools/msa/clustalw2/)^61^,^62^. Next, the consensus was created with equal or stereochemically similar amino acid residues in the same aligned position. For stereochemically identical residues we used the symbol “h” for hydrophobic, “+” for cationic, “–” for anionic and “Z” for E or Q residues (according to the International Union for Pure and Applied Chemistry one-letter amino acid code^63^).

### Structure comparison between APOE and PLIN3

Protein structures coordinates were extracted from the Protein Data Bank (PDB, www.pdb.org). All structures are from apoproteins (*i.e.*, without bound non-protein components) and PDB identification codes are specified ahead after the protein name. APOE protein structures from human and mouse (hAPOE, 2L7B^38^; mAPOE, 1YA9^64^) were used in the analysis. The 2L7B PDB file structure of hAPOE was obtained from nuclear magnetic resonance (NMR) data^38^. It was selected out of 14 hAPOE available structures since it is currently the most complete structure available, obtained from the full length protein^38^. This PDB file contains 20 model structures of minimal energy that fit the experimental results, with little variation between each other (average Cα RMSD of 1.35 Å). Given this high resemblance between conformers, the first model (model 1) was chosen as the hAPOE reference structure, since it was also described by the authors as the best representative conformer in the ensemble^38^. 1YA9 is an X-ray structure of mAPOE (residues P4 to V177), and the only presently available^64^. Regarding PLIN3, the mouse protein X-ray structure (residues N206 to T430), with a single conformer (mPLIN3, 1SZI^39^) is presently the only PLIN3 structure available.

These three protein structures were superimposed through UCSF Chimera 1.6.2 software^65^ MatchMaker tool^66^. After that, we carefully analyzed the superposition visually. Then, using the Match-Align tool of UCSF Chimera^66^, which returns a sequence alignment based on the regions closer in space (less than 5 Å) and taking into account the structure superimposition, we identified the residues simultaneously similar in structure and sequence (*e.g.*, revealing shared amino acid motifs at equivalent structural positions). Protein structure figures were obtained using UCSF Chimera 1.6.2^65^. Structure alignments were complemented by calculating the amino acid residues α-carbons root-mean-squared deviation (Cα RMSD) in the Swiss-PdbViewer 4.1 software^67^, using the equation 1:


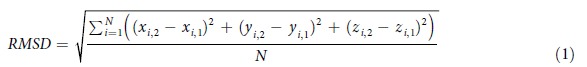


where *x*, *y* and *z* are the Cartesian coordinates of each atom in the aligned position, *N* is the number of atom pairs compared between structures 1 (reference) and 2, and *i* is the index of each atom in the set of comparison. To evaluate the degree of variation within the hAPOE structure that serves as a generic measure of the variability between the protein conformers, we averaged the Cα RMSD between model 1 and the other 19 hAPOE models.

### Structure and sequence comparison between DENV C and hAPOEαN

We compared the hAPOEαN (residues K1 to S44) structure from the 2L7B file (model 1) with the DENV serotype 2 capsid protein structure (1R6R), previously obtained from NMR data^27^, which has 21 minimal energy models deposited in the PDB. Model 21, an energy-minimized average of several NMR conformers which serves as the best conformer to the data, was used as DENV C reference structure for the analysis. DENV C and hAPOEαN comparison was performed via the MatchMaker tool^66^ of UCSF Chimera 1.6.2^65^. Then, we compared the amino acid sequence of the superimposing regions of both proteins via the Match-Align tool of UCSF Chimera^66^, with residue proximity of less than 5 Å. The sequence consensus was constructed taking into account stereochemical similarities between amino acid residues aligned in the same position, as described above. RMSD values were calculated via Swiss-PdbViewer 4.1^67^ and figures were constructed using UCSF Chimera 1.6.2^65^.

### Consensus motifs search

For the analysis of the consensus sequences of APOEα4 and PLIN3α5 (Table 1), and of hAPOEαN and DENV C α1-α2 regions (Table 2), we used the ScanProsite web server from ExPASy (http://prosite.expasy.org/scanprosite^45^), scanning the motifs against the UniProtKB / Swiss-Prot protein sequence database (which contained 540,732 protein sequences deposited, including isoforms, when the analysis was performed). Motifs were based on the consensus sequences. PROSITE pattern syntax (http://prosite.expasy.org/scanprosite/scanprosite_doc.html#scanning_options) was used to describe both the motifs and the stereochemical similarities between amino acid residues. For each motif, we registered the number of hits from our proteins of interest (in different organisms), the number of results given and the expected number of random matches in a 100,000 protein sequences database. As the database used contained 540,732 protein sequences, we corrected accordingly (multiplying the given random match estimate by 5.41).

### AFM-based force spectroscopy inhibition assay

To evaluate if the APOE at the surface of VLDL is involved on the binding to DENV C, we performed AFM-based force spectroscopy measurements of DENV C (VCPBio, Shenzhen, China) binding to VLDL (Kalen Biomedical LLC, Montgomery Village, MD, USA) in the presence or absence of a rabbit anti-human APOE antibody (Sigma, St. Louis, MO, USA), raised against a recombinant fragment corresponding to APOE amino acid residues 1 to 129. The effects of anti-APOE were evaluated by force spectroscopy after incubating the VLDL sample with the antibody at 100 μg/L and 1 μg/L, for 1 h at room temperature. All measurements were conducted in TEE-KCl buffer pH 7.4 (20 mM Tris-HCl, 100 mM KCl, 1 mM EDTA and 1 mM EGTA). Force spectroscopy measurements were performed on a NanoWizard II atomic force microscope (JPK Instruments, Berlin, Germany), mounted on an Axiovert 200 inverted optical microscope (Zeiss, Jena, Germany), using DENV C functionalized OMCL TR-400-type silicon nitride AFM tips (Olympus, Tokyo, Japan), as previously described^30^,^31^,^68^. The spring constant of the tips was calibrated by the thermal fluctuation method. The applied force was adjusted to 200 pN before retraction. Molecular recognition was searched by intermittently pressing the cantilevers on each VLDL, adsorbed on freshly cleaved muscovite mica. Data collection for each force-distance cycle was performed at 2 μm/s, leading to a loading rate of 4 nN/s. Force curves were analyzed using the JPK image processing software v.4.2.61. For each experimental condition, approximately 5000 force-distance curves were collected, analyzed and adjusted by polynomial fit. Each experiment was performed at least three times, each time on different VLDL samples and with different functionalized tips. Histograms of the (un)binding forces of each data set were constructed choosing the ideal bin size to achieve the best fit (6 pN). Force rupture values below 10 pN were considered to represent noise, artifacts or unspecific interactions. From each histogram, the most likely single DENV C-VLDL binding rupture force was determined fitting the distributions of the rupture forces with the Gaussian model. The maximum values of the Gaussian peaks represent a single-molecule-based statistical measure of the force of the molecular bond. The anti-APOE antibody was purchased from Sigma-Aldrich (St. Louis, MO, USA). As a negative control for the antibody-blocking experiments, the effect of the presence of an unspecific isotype-matched antibody (normal rabbit IgG serum; Santa Cruz Biotechnology, Santa Cruz, CA, USA) was tested at 100 μg/L and at 1 μg/L, being incubated with VLDL for 20 minutes at room temperature prior to the AFM measurements.

## Additional Information

**How to cite this article**: Faustino, A. F. *et al.* Understanding Dengue Virus Capsid Protein Interaction with Key Biological Targets. *Sci. Rep.* **5**, 10592; doi: 10.1038/srep10592 (2015).

## Supplementary Material

Supplementary Information

## Figures and Tables

**Table 1:** Validation of APOE α-helix 4 and PLIN3 α-helix 5 sequence consensus.

Motif ID	Proposed 23 amino acid residues motifs	Total hits found	APOE hits	PLIN3 hits	Other protein hits	Random hits per 100 k sequences	Expected random hits
AP1	[ALVIM]-X-[STCNQDEH]-[STCNQDEH]-[ALVIM]-[RK]-X(10)-[STCNQDEH]-D-L-Q-X(2)-[ALVIMFYW]	40	16	6	18	2.1	11
AP2	[ALVIMFYW]-X-[ST]-[NH]-[ALVIMFYW]-[RK]-X(10)-[NQDE]-D-L-Q-X(2)-[ALVIMFYW]	25	19	6	0	0.12	1
AP3	[ALVIM]-X-[ST]-[NH]-[ALVIM]-[RK]-X(10)-[NQDE]-D-L-Q-X(2)-[ALVIMFYW]	22	16	6	0	0.073	0
AP4	[ALVIM]-X-[ST]-[NH]-[ALVIM]-[RK]-X(10)-[NDE]-D-L-Q-X(2)-[ALVIMFYW]	22	16	6	0	0.058	0
AP5	L-X-[ST]-[NH]-[ALVIM]-[RK]-X(10)-[NDE]-D-L-Q-X(2)-[ALVIMFYW]	22	16	6	0	0.017	0
AP6	L-X-[ST]-[NH]-[LVI]-[RK]-X(10)-[NDE]-D-L-Q-X(2)-[ALVIMFYW]	22	16	6	0	0.012	0
AP7	L-X-[ST]-[NH]-[LVI]-[RK]-X(10)-[NDE]-D-L-Q-X(2)-[LF]	22	16	6	0	0.0040	0
AP8	L-X-[ST]-[NH]-[LV]-[RK]-X(10)-[NDE]-D-L-Q-X(2)-[LF]	22	16	6	0	0.0029	0

**Table 2:** Validation of APOE α-helix N2 and Flavivirus C proteins α-helix 2 sequence consensus.

**Motif ID**	**Proposed 13 amino acid residues motifs**	**Total hits found**	**APOE hits**	**DENV C hits**	**Other** ***Flavivirus*** **hits**	**Other protein hits**	**Random hits per 100 k sequences**	**Expected random hits**
AF1	[RK]-[ALVIMFYW]-X-[LM]-A-[ALVIMFYW]-X(2)-F-[ALVIMFYW]-[STCNQDEHRK]-[FVY]-X	61	4	22	7	28	8.6	47
AF2	[RK]-[ALVIMFYW]-X-[LM]-A-[ALVIMFYW]-X(2)-F-[LFW]-[STCNQDEHRK]-[FVY]-X	42	4	22	7	9	3.2	17
AF3	[RK]-[FLIVMW]-X-[LM]-A-[FLIVM]-X(2)-F-[LFW]-[STCNQDEHRK]-[FVY]-X	36	4	22	7	3	1.6	9
AF4	[RK]-[FLIVMW]-X-[LM]-A-[FLIVM]-X(2)-F-[LFW]-[DERK]-[FVY]-X	34	4	22	7	1	0.76	4
AF5	[RK]-[FLIVMW]-X-[LM]-A-[FLIVM]-X(2)-F-[LFW]-[DRK]-[FVY]-X	34	4	22	7	1	0.55	3
AF6	[RK]-[FLIVMW]-X-[LM]-A-[FLIVM]-X(2)-F-[LFW]-[DRK]-[FVY]-[LT]	33	4	22	7	0	0.084	0
AF7	[RK]-[FLIVMW]-X-[LM]-A-[FLIVM]-X(2)-F-[LFW]-[DRK]-[FVY]-L	26	4	22	0	0	0.053	0
AF8	[RK]-[LMW]-X-[LM]-A-[FL]-X(2)-F-[LFW]-[DRK]-[FVY]-L	26	4	22	0	0	0.011	0
