# Supplementary material for: Understanding Dengue Virus Capsid Protein Interaction with Key Biological Targets
Source: Sci Rep. 2015 Jul 10;5:10592. doi: 10.1038/srep10592 (PMC4498383; doi:10.1038/srep10592)
Supplement: Supplementary Information [file srep10592-s1.pdf]

Supplementary Information

Title

Understanding Dengue Virus Capsid Protein Interaction with Key Biological Targets

Authors

André F. Faustino[1#], Ivo C. Martins[1#]*, Filomena A. Carvalho[1], Miguel A. R. B. Castanho[1], Sebastian Maurer-Stroh[2, 3], Nuno C. Santos[1]*

Affiliations

[1] – Instituto de Medicina Molecular, Faculdade de Medicina, Universidade de Lisboa, Lisbon, Portugal.

[2] – Bioinformatics Institute (BII), Agency for Science, Technology and Research (A*STAR), Singapore.

[3] – School of Biological Sciences (SBS), Nanyang Technological University (NTU), Singapore.

# – These authors contributed equally to the work.

* – Corresponding authors

Nuno C. Santos – nsantos@fm.ul.pt

Ivo C. Martins – ivomartins@fm.ul.pt

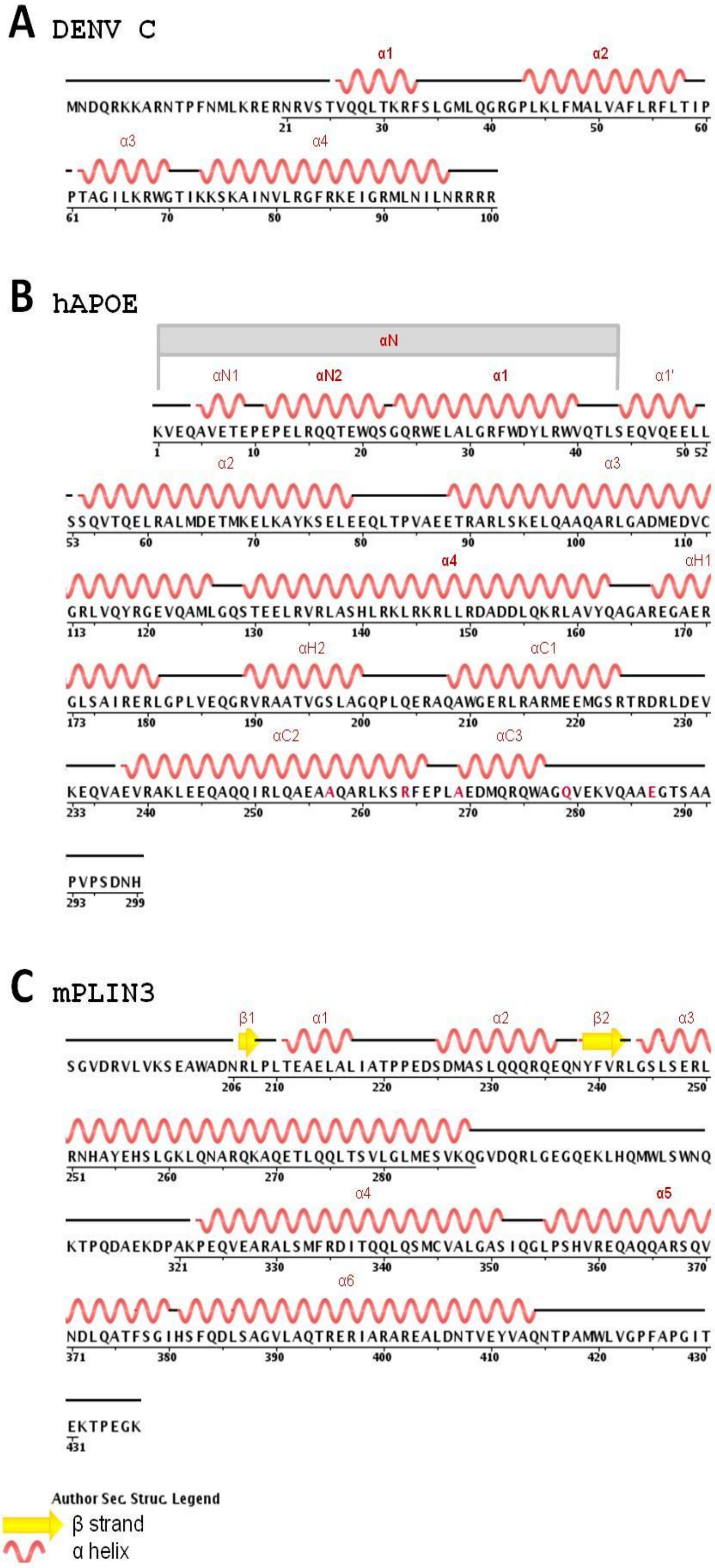

Supplementary Figure S1 | DENV C, hAPOE and mPLIN3 secondary structure nomenclature. Nomenclature of the secondary structure domains of (A) DENV C, (B) hAPOE and (C) mPLIN3 proteins, according to the secondary structure author assignment of the respective 1R6R, 2L7B and 1SZI PDB files. In (B), the segment hAPOEαN described in the manuscript, composed by the N-terminal hAPOE residues 1 to 44, is identified by the gray box.

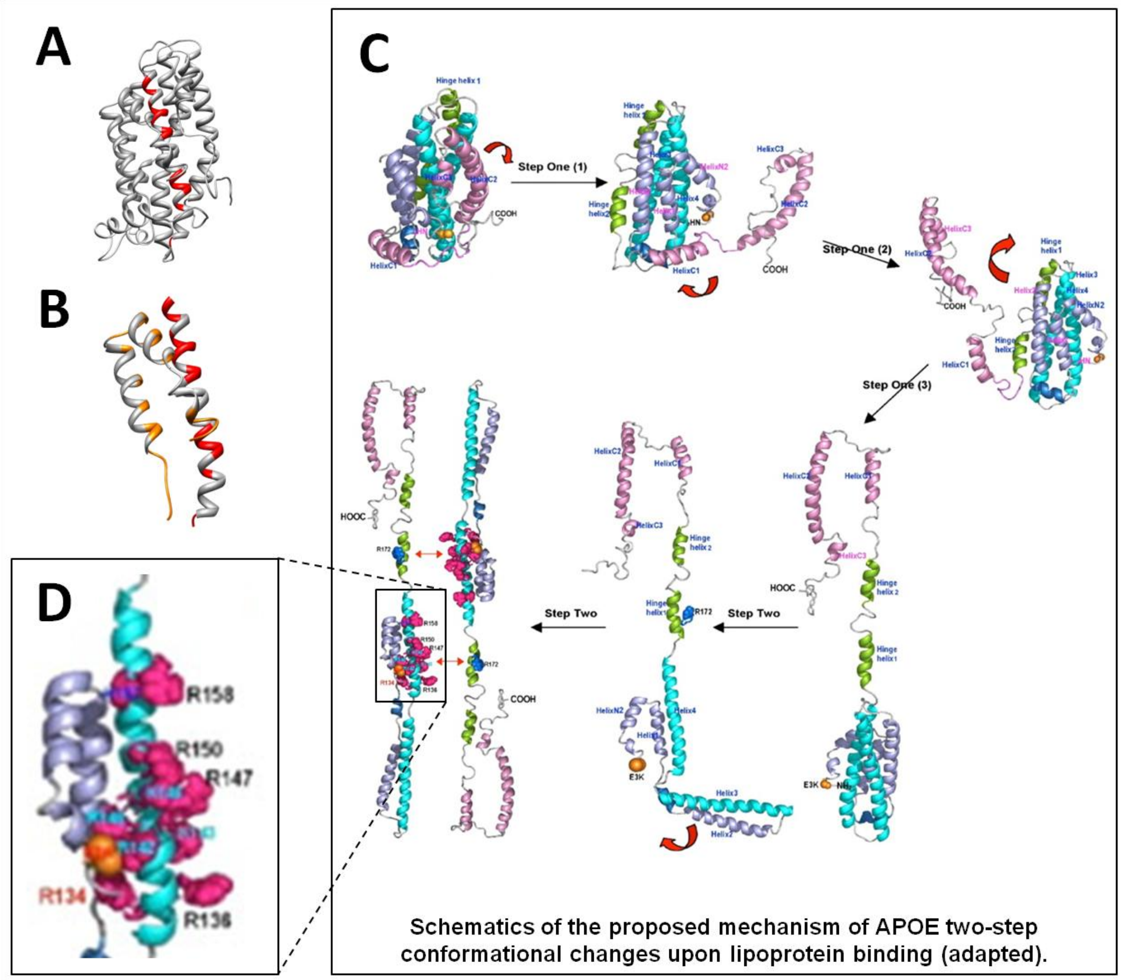

Supplementary Figure S2 | hAPOE α-helix 4 (hAPOEα4) interacts directly with the hAPOE α-helical N-terminal region (hAPOEαN).(A) hAPOE ribbon structure (PDB ID: 2L7B) with the hAPOEα4 residues conserved between APOE and PLIN3 highlighted in red. (B) Display as indicated in (A), but showing only the hAPOEαN and hAPOEα4 sections, with the hAPOEαN amino acid residues within 5 Å from hAPOEα4 colored in orange. (C) The hAPOE structure undergoes a conformational rearrangement upon interacting with lipoproteins (adapted with permission from Chen, J., Li, Q. & Wang. J. *Proc. Natl. Acad. Sci. USA* **108**, 14813-14818 (2011)). (D) Even within this rearranged structure, hAPOEαN and hAPOEα4 remain bound.
